# What patients do when they feel dizzy: behavioural signatures reveal diagnostic clues in common vestibular disorders

**DOI:** 10.1007/s00415-026-14133-0

**Published:** 2026-09-10

**Authors:** Huseyin Nezih Ozdemir, Iryna Klopotovska, Diego Kaski

**Affiliations:** 1 SENSE Research Unit, Department of Clinical and Movement Neurosciences, UCL, London, WC1N 3BG UK; 2 Department of Neurology, Ege University Medical School, İzmir, 35100 Türkiye

**Keywords:** Dizziness, Vestibular disorders, Behavioural adaptation, Avoidance behaviour, Diagnostic biomarkers

## Abstract

**Background:**

Diagnosis of vestibular disorders relies heavily on clinical history, yet how patients adapt their behaviour in response to dizziness remains relatively unexplored. We investigated whether adopted avoidance behaviours and coping strategies provide additional diagnostic information and insights into disease mechanisms.

**Methods:**

Consecutive patients attending a specialist dizziness clinic underwent a structured interview before clinical assessment. Participants reported dizziness triggers, avoidance behaviours, and coping responses. Final diagnoses were established independently by a vestibular neurologist, and associations between behavioural patterns and diagnosis were examined using logistic regression.

**Results:**

One hundred and nine patients were included (mean age 55.3 years; 71.5% female), including thirty-three with vestibular migraine (VM), thirty-three with persistent postural-perceptual dizziness (PPPD), fourteen with benign paroxysmal positional vertigo (BPPV), and fifteen with orthostatic dizziness (OD). Reading whilst travelling predicted VM (OR 20.99), visually stimulating environments predicted PPPD (OR 12.45), head movements predicted BPPV (OR 11.51) and standing quickly was associated with OD. Avoidance and coping behaviours were similarly diagnosis-specific, including avoidance of bright or noisy environments and resting in dark or quiet settings in VM, avoidance of visually complex environments and maintaining activity in PPPD, and avoidance of head movements and remaining still during attacks in BPPV; OD patients avoided sudden standing.

**Conclusion:**

Patients with common causes of dizziness exhibit distinct behavioural signatures that reflect the underlying mechanisms of their disorders. These intuitive adaptations may provide valuable clinical clues beyond conventional symptom descriptions. Questions about avoidance behaviours and coping strategies may improve diagnostic accuracy and understanding of vestibular disorders.

**Supplementary Information:**

The online version contains supplementary material available at https://doi.org/10.1007/s00415-026-14133-0.

## Introduction

Dizziness is one of the most prevalent neurological symptoms worldwide. It accounts for 1.0% to 15.5% of visits in primary care settings and is amongst the most common reasons for neurological consultations [1, 2]. It is associated with substantial disease burden and can significantly impair quality of life [3].

Despite the high prevalence of dizziness, definitive confirmatory tests are lacking for many vestibular disorders, and neuroimaging has limited diagnostic utility in most cases of dizziness. Consequently, diagnosis often relies heavily on clinical evaluation. However, patients frequently struggle to describe their symptoms precisely [4]. For that reason, careful history taking and clinical examination are essential for establishing an accurate diagnosis, particularly in patients with recurrent or chronic syndromes [5].

Current diagnostic approaches concentrate on identifying immediate triggers, prominent associated symptoms, and timing during history-taking [5]. Nevertheless, many aspects of the patient’s experience remain underexplored, particularly how individuals respond to dizziness in their daily lives. The identification of clinical symptoms and behavioural responses to dizziness may facilitate earlier diagnosis, reduce delays in treatment initiation and prevent progression to chronic dizziness [6]. Furthermore, behavioural patterns adopted by patients may reflect underlying disease mechanisms and provide additional diagnostic clues beyond symptom descriptions alone.

This study focussed on identifying specific precipitators, avoidance behavioural, and behavioural coping strategies, with the aim of exploring whether these unintentional patterns provide additional clinical clues and insights into the pathophysiology of common causes of dizziness encountered in a vestibular clinic.

## Methods

This was an exploratory observational study designed to identify disorder-specific behavioural patterns amongst patients with dizziness. Consecutive patients presenting with dizziness to a specialised dizziness clinic at the National Hospital for Neurology and Neurosurgery, London, UK, between April 2025 and April 2026 underwent a structured interview as part of the study. Participants were eligible for inclusion if they were aged 18 years or older, resided in the UK and were able to communicate in English.

Interviews were conducted before the clinical consultation by one of two researchers (HNO or IK), prior to diagnostic assessment. The structured interview comprised ten predefined questions relating to symptom characteristics, associated features and behavioural responses. Responses were recorded according to prespecified response categories. Questions addressing precipitating factors, avoidance behaviours, behavioural responses and temporal characteristics in patients with dizziness were selected for analysis, as these were considered most directly relevant to the study’s focus on behavioural adaptation. The present analysis focussed on responses to questions addressing (1) temporal characteristics, comprising dizziness pattern (“How does your dizziness usually occur?”) and duration (“How long does each dizziness episode usually last?”), (2) precipitating factors (“What things make your dizziness worse or bring it on?”), (3) avoidance behaviours (“Do you avoid doing anything because it makes your dizziness worse or you are afraid of triggering it?”) and (4) behavioural responses (“What do you usually do when you feel dizzy?”). The full interview instrument is provided in the Supplementary Material.

Following the interview, patients underwent routine clinical assessment by a neurologist specialising in vestibular disorders (DK), who was blinded to the interview responses. Final diagnoses were made based on the clinical evaluation and relevant investigations. Established diagnostic criteria, including Bárány Society and International Classification of Headache Disorders criteria where applicable [7, 8], were used when making the diagnosis.

Descriptive statistics were used to summarise demographic characteristics. Age was reported as mean and standard deviation (SD), whereas sex was reported as frequency and percentage. Patients were classified into six diagnostic groups reflecting the commonest diagnoses: vestibular migraine (VM), persistent postural-perceptual dizziness (PPPD), benign paroxysmal positional vertigo (BPPV), orthostatic dizziness (OD), other peripheral vestibular disorders (e.g. Ménière disease, vestibular neuritis, and bilateral vestibular failure) and central structural disorders (e.g. posterior circulation stroke and spinocerebellar ataxia). For each diagnostic group, a binary outcome variable was created indicating the presence or absence of the condition with diagnosis serving as the dependent variable in all regression models. For temporal characteristics (Question 2 and Question 3), each response category was mutually exclusive by design, making joint multivariable entry statistically inappropriate due to perfect collinearity with the model intercept; each category was therefore tested individually against each diagnosis using univariable binary logistic regression. Separate multivariable binary logistic regression models were constructed for each diagnostic group and for each of the three multi-select questions analysed (Questions 5, 9, and 10), with the response options within each question entered as independent variables.

To enhance clinical interpretability, responses that demonstrated significant associations with a diagnosis in the regression analyses were further evaluated using 2 × 2 contingency tables. Sensitivity, specificity, positive predictive value (PPV) and negative predictive value (NPV) were calculated. Statistical analyses were performed using IBM SPSS Statistics version 26.0 (IBM Corp., Armonk, NY, USA). A *p* value <0.05 was considered statistically significant.

## Results

A total of 109 participants were included in the study. The diagnostic distribution was as follows: 33 patients (30.2%) with VM, 33 (30.2%) with PPPD, 15 (13.7%) with OD, 14 (12.8%) with BPPV, 8 (7.7%) with central structural disorders, and 6 (5.5%) with other peripheral vestibular disorders. The central structural disorders included six patients with posterior circulation stroke, one with mild traumatic brain injury, and one with Chiari type I malformation with syringomyelia. The other peripheral vestibular disorders included four patients with bilateral vestibular loss and two with acute unilateral peripheral vestibulopathy.

Of the 109 participants, 78 (71.5%) were female and 31 (28.4%) were male. The mean age was 55.3 years (SD = 16.7). Supplementary Table S1 provides detailed information on age and sex distribution by diagnostic group.

**Fig. 1:**
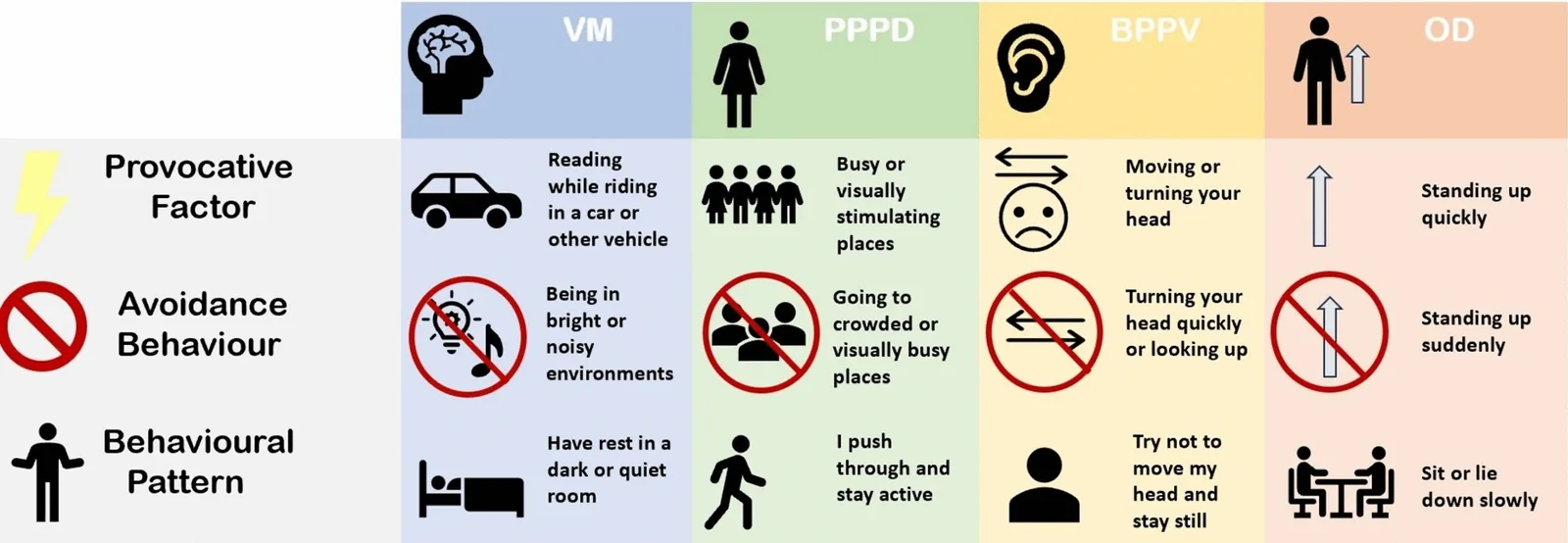
Disease-specific behavioural patterns in common causes of dizziness. The figure summarises the precipitating factors, avoidance behaviours and behavioural responses most strongly associated with vestibular migraine (VM), persistent postural-perceptual dizziness (PPPD), benign paroxysmal positional vertigo (BPPV) and orthostatic dizziness (OD). The precipitating factor “standing up ” and the behavioural response “sit or lie down slowly” were more frequently reported by patients with OD; however, neither association reached statistical significance in logistic regression analysis

Logistic regression analyses identified distinct disorder-specific associations for temporal characteristics, precipitating factors, avoidance behaviours and behavioural responses in VM, PPPD, BPPV and OD (Table 1). An infographic summarising the disease-specific behavioural signatures observed in our cohort is presented in Fig. 1. Supplementary Table S2 provides detailed interview results, including response frequencies within diagnostic and non-diagnostic groups, together with sensitivity, specificity, positive predictive value (PPV) and negative predictive value (NPV) analyses. The full logistic regression models for each diagnostic group for multi-select questions and univariable logistic regression results for one-select questions are presented in Supplementary Material S3.

**Table 1:** Key structured interview items and response patterns associated with common causes of dizziness across precipitating factors, avoidance behaviours and behavioural responses

Aspect	Question	Answer pattern	Associated diagnosis	OR (95% CI)	*p* value
Temporal pattern	How does your dizziness usually occur?	Comes and goes repeatedly	VM	3.04 (1.04–8.86)	0.041
		Continuous, all the time	PPPD	7.04 (2.36–20.93)	<0.001
		Comes and goes repeatedly	BPPV	4.64 (0.56–38.29)	0.144
		Comes and goes repeatedly	OD	2.03 (0.53–7.76)	0.299
Duration	How long does your dizziness usually last?	Minutes to hours	VM	1.45 (0.60–3.50)	0.401
		Days or longer	PPPD	2.81 (1.16–6.80)	0.022
		Seconds to minutes	BPPV	6.74 (1.35–33.56)	0.020
		Seconds to minutes	OD	1.76 (0.58–5.29)	0.312
Precipitating factor	What things make your dizziness worse or bring it on?	Reading whilst riding in a car or other vehicle	VM	20.99 (6.30–69.92)	<0.001
		Busy or visually stimulating places	PPPD	12.45 (3.98–38.88)	<0.001
		Moving or turning your head	BPPV	11.51 (1.30–101.88)	0.028
		Standing up quickly	OD	3.59 (0.93–13.83)	0.062
Avoidance behaviour	Do you avoid doing anything because it makes your dizziness worse or you are afraid of triggering it	Being in bright or noisy environments	VM	8.20 (2.33–28.87)	0.001
		Going to crowded or visually busy places	PPPD	4.55 (1.51–13.63)	0.007
		Turning your head quickly or looking up	BPPV	9.39 (1.75–50.40)	0.009
		Standing up suddenly	OD	3.94 (1.004–15.48)	0.049
Behavioural response	What do you usually do when you feel dizzy?	Have rest in a dark or quiet room	VM	5.89 (2.11–16.46)	0.001
		I push through and stay active	PPPD	6.46 (1.51–27.60)	0.012
		Try not to move my head and stay still	BPPV	4.60 (1.02–20.60)	0.046
		Sit or lie down slowly	OD	2.61 (0.71–9.60)	0.146

## Discussion

This report explored temporal patterns, precipitating factors, avoidance behaviours and behavioural responses amongst patients with dizziness. These patterns may provide pathophysiological insights and offer additional clinical clues that supplement established diagnostic criteria for VM, PPPD, BPPV, OD.

The temporal characteristics of the disease were generally consistent with the literature. Episodic dizziness was associated with VM (*p* = 0.041), as is noted in the literature [9]. Patients with PPPD were considerably more likely to report continuous dizziness (*p* < 0.001), in line with its underlying chronicity [6, 11]. Although patients with BPPV and OD tended to report dizziness that “comes and goes” (BPPV: *p* = 0.144; OD: *p* = 0.299), this did not reach significance, likely reflecting the relatively small number of participants in these groups. Patients with PPPD were more likely to report dizziness lasting days or longer, again reflecting the chronic nature of the disorder as it was in the temporal pattern (*p* = 0.022) [6, 11]. Patients with BPPV were more likely to report episodes lasting seconds to minutes, consistent with its brief, paroxysmal presentation (p = 0.020). No clear duration category emerged for VM, likely reflecting its diagnostic duration criterion, which spans more than one of our response categories.

Consistent with previous reports, reading whilst travelling as a passenger in a vehicle emerged as a precipitating factor in VM [10]. This may reflect the heightened susceptibility to sensory conflict underlying VM and have predictive value for the diagnosis of VM in clinical practice [10]. Visually stimulating environments are a well-known precipitating factor in PPPD [6, 11]. Head turning or positional changes are commonly reported triggers of BPPV, consistent with the abnormal movement of displaced otoconia within the semi-circular canals [12]. Consistent with this mechanism, patients with BPPV in our cohort were significantly more likely to report symptom provocation by head movements. Symptoms provoked by standing up quickly are characteristic of OD and are thought to result from transient cerebral hypoperfusion during orthostatic stress [13].

Avoidance behaviours are common in patients with dizziness [14]. Their recognition may aid diagnosis and help identify behaviours that contribute to disability [14]. In our cohort, avoidance of bright or noisy environments was associated with VM, consistent with the heightened visual and auditory sensitivity that characterises migraine and VM [15]. Avoidance of crowded or visually busy places was associated with PPPD, reflecting the increased reliance on visual cues for spatial orientation and hypersensitivity to complex visual stimuli that are characteristic of PPPD [6]. Patients with BPPV frequently reported avoiding quick head movements to prevent vertigo provoked by abnormal stimulation of the affected semicircular canal by displaced otoconia [12]. Avoiding standing up quickly was associated with OD, representing a strategy to prevent symptoms precipitated by orthostatic stress and transient cerebral hypoperfusion [13].

Patients with dizziness often adopt adaptive strategies to cope with symptoms and minimise their impact on daily activities [3, 15]. Such adaptations may include planning daily activities, selecting environments perceived as safer, and employing behavioural responses to dizziness [3, 15]. In our cohort, patients from different diagnostic groups adopted distinct behavioural patterns to manage dizziness. Resting in a dark or quiet room was associated with VM, likely as a coping strategy for the visual and auditory hypersensitivity associated with VM [16]. Patients with PPPD frequently reported trying to stay active, reflecting an effort to stay active but perhaps also a reflection of the perceptual nature of the symptoms, being physically able to perform daily activities despite persistent symptoms. Patients with BPPV frequently reported trying not to move their head and remaining still, an adaptive response to recurrent positionally triggered vertigo. Patients with OD more often reported sitting or lying down slowly, potentially as a strategy to counteract symptoms arising from orthostatic intolerance and reduce the risk of falls, although this association was not statistically significant. The lack of statistical significance may reflect the relatively small number of patients with OD in our cohort, or perhaps that behavioural responses to light-headedness are indeed more heterogeneous.

These findings may also have practical implications for the clinical assessment of patients with dizziness. Questioning about precipitating factors, avoidance behaviours and behavioural responses can capture aspects of the patient’s experience that remain underexplored in routine history-taking. Notably, “reading while riding in a car or other vehicle” was strongly predictive of VM (OR 20.99), consistent with prior work showing that motion sickness whilst reading in a moving vehicle discriminates VM from other dizziness diagnoses [10, 17]. Beyond diagnosis, understanding these behavioural patterns may help clinicians appreciate their impact on patients’ daily and occupational functioning: triggers such as bright environments in VM and awkward head positions in BPPV have been reported to interfere with work-related tasks, contributing to reduced productivity and, at times, absenteeism [15]. Although patients with PPPD often reported “pushing through and staying active,” this coping strategy does not preclude a significant impact on daily life. Future studies focussing on precipitating factors, avoidance behaviours and behavioural responses in larger cohorts are needed to confirm their diagnostic value and their effect on patients’ functioning.

The current report has several limitations. The structured interview used was not a validated instrument, which limits direct comparability with other studies. Subgroup sizes for some diagnoses were small, limiting statistical power and precluding stable effect estimates for several response categories. This was also a single-centre study, which may limit the generalisability of our findings.

Taken together, participants reported distinct precipitants, avoidance behaviours and behavioural responses associated with common causes of dizziness. These patterns appear closely related to the underlying pathophysiology of the respective disorders. Notably, patients adopted these behaviours without formal knowledge of the disease mechanisms, suggesting that they represent intuitive adaptations developed through repeated symptom experiences. Greater attention to these behavioural patterns may provide insights into disease pathophysiology and offer additional clinical clues during diagnostic assessment.

## Supplementary Information

Below is the link to the electronic supplementary material.

Supplementary file1 (DOCX 17 kb)

Supplementary file2 (DOCX 21 kb)

Supplementary file3 (DOCX 76 kb)

## Data Availability

The datasets generated and/or analysed during the current study are available from the corresponding author on reasonable request.
