# Supplementary material for: What patients do when they feel dizzy: behavioural signatures reveal diagnostic clues in common vestibular disorders
Source: J Neurol. 2026 Sep 10;273(10):587. doi: 10.1007/s00415-026-14133-0 (PMC13562255; doi:10.1007/s00415-026-14133-0)
Supplement: Supplementary file 1 [file 415_2026_14133_MOESM1_ESM.docx]

**Dizziness Interview Form**

**1. What best describes your dizziness?**

**a)** Spinning sensation (like the room or surroundings are moving)	**b)** Lightheadedness or feeling faint

**c)** Feeling unsteady or off-balance

**2. How does your dizziness usually occur?**

**a)** Suddenly, has happened once	**b)** Comes and goes repeatedly

**c)** Continuous, all the time

**3. How long does each dizziness episode usually last?**

**a)** Seconds to minutes	**b)** Minutes to hours	**c)** Days or longer

**4. What other symptoms, if any, do you experience when you feel dizzy?** *(Choose all that apply)*

**a)** Face weakness or numbness, or difficulty speaking	**b)** Vision change or trouble swallowing

**c)** Weakness or numbness on one side of your body	**d)** Loss of balance and poor coordination

**e)** None of these

**5. What things make your dizziness worse or bring it on?** *(Choose all that apply)*

**a)** Moving or turning your head (like looking up or rolling over in bed)

**b)** Standing up quickly

**c)** Busy or visually stimulating places (like crowded areas or places with moving patterns)

**d)** Feeling anxious or stressed

**e)** Reading while riding in a car or other vehicle

**f)** None of these

**6. Are you currently taking any of these medicines?** *(Choose all that apply)*

**a)** Blood pressure or water pills	**b)** Anti-depressants or sedatives

**c)** Pain medicines (like opioids or nerve pain medications)	**d)** None of these

**7.** **Do you currently have, or have you recently experienced, any other symptoms or health problems that you think may be related to your dizziness?** *(Choose all that apply)*

**a)** Headache, nausea or sensitivity to light or sound

**b)** Hearing loss or feeling of fullness or pressure in the ear that comes and goes

**c)** Falls or almost falling

**d)** Recent head trauma (e.g., from a fall, accident, or blow to the head)

**e)** None of these

**8. Have you ever fallen?**

**a)** I have neither felt unsteady nor fallen

**b)** I feel unsteady but never have fallen

**c)** When I stand up quickly, I feel like I need to hold onto something

**d)** I have fallen, or others have noticed I am unsteady or off-balance

**9.** **Do you avoid doing anything because it makes your dizziness worse or you are afraid of triggering it?** *(Choose all that apply)*

**a)** Driving	**b)** Going to crowded or visually busy places

**c)** Exercising or walking long distances	**d)** Turning your head quickly or looking up

**e)** Standing up suddenly	**f)** Being in bright or noisy environments

**g)** Other: ___________	**h)** None of these

**10. What do you usually do when you feel dizzy?** *(Choose all that apply)*

**a)** Have rest in a dark or quiet room	**b)** Try not to move my head and stay still

**c)** Hold on to something or lean against a wall	**d)** Sit or lie down slowly

**e)** Focus on breathing or try to stay calm	**f)** I push through and stay active

**g)** Other: ___________	**h)** None of these
