# Supplementary material for: What patients do when they feel dizzy: behavioural signatures reveal diagnostic clues in common vestibular disorders
Source: J Neurol. 2026 Sep 10;273(10):587. doi: 10.1007/s00415-026-14133-0 (PMC13562255; doi:10.1007/s00415-026-14133-0)
Supplement: Supplementary file 2 [file 415_2026_14133_MOESM2_ESM.docx]

**Supplementary Table 1.** Demographic characteristics of participants according to diagnostic group.

|  | **VM**  N = 33 | **PPPD**  N = 33 | **BPPV**  N = 14 | **OD**  N = 15 | **Central Structural**  N = 8 | **Other Peripheral Vestibular**  N = 6 |
| --- | --- | --- | --- | --- | --- | --- |
| Age, years, mean (SD) | 45.0 (14.4) | 56.8 (15.7) | 67.9 (12.6) | 66.6 (12.6) | 53.2 (19.9) | 53.2 (13.1) |
| Sex, female, n (%) | 28 (84.8) | 26 (78.7) | 9 (64.2) | 8 (53.3) | 5 (67.5) | 4 (66.6) |

**Supplementary Table 2.** Sensitivity, specificity, positive predictive value, and negative predictive value of key structured interview responses associated with common causes of dizziness.

| **Aspect** | **Answer** | **Diagnosis** | **Diagnosis group n/N** | **Other patients n/N** | **Sensitivity %** | **Specificity %** | **PPV %** | **NPV %** |
| --- | --- | --- | --- | --- | --- | --- | --- | --- |
| Precipitating factor | Reading while riding in a car or other vehicle | VM | 20/33 | 7/76 | 61 | 91 | 74 | 84 |
|  | Busy or visually stimulating places | PPPD | 23/33 | 20/76 | 70 | 74 | 54 | 85 |
|  | Moving or turning your head | BPPV | 13/14 | 51/95 | 93 | 46 | 20 | 98 |
|  | Standing up quickly | OD | 11/15 | 51/94 | 73 | 46 | 18 | 92 |
| Avoidance behaviour | Being in bright or noisy environments | VM | 14/33 | 13/76 | 42 | 83 | 52 | 77 |
|  | Going to crowded or visually busy places | PPPD | 17/33 | 16/76 | 52 | 79 | 52 | 79 |
|  | Turning your head quickly or looking up | BPPV | 12/14 | 43/95 | 86 | 55 | 22 | 96 |
|  | Standing up suddenly | OD | 9/15 | 38/94 | 60 | 60 | 19 | 90 |
| Behavioural response | Have rest in a dark or quiet room | VM | 15/33 | 9/76 | 46 | 88 | 63 | 79 |
|  | I push through and stay active | PPPD | 16/33 | 14/76 | 49 | 82 | 53 | 79 |
|  | Try not to move my head and stay still | BPPV | 11/14 | 36/95 | 79 | 61 | 23 | 95 |
|  | Sit or lie down slowly | OD | 11/15 | 45/94 | 73 | 52 | 20 | 93 |

**Supplementary Table 3.** Sensitivity, specificity, positive predictive value, and negative predictive value of additional structured interview responses (temporal pattern and duration) associated with common causes of dizziness.

| **Aspect** | **Answer** | **Diagnosis** | **Diagnosis group n/N** | **Other patients n/N** | **Sensitivity %** | **Specificity %** | **PPV %** | **NPV %** |
| --- | --- | --- | --- | --- | --- | --- | --- | --- |
| Temporal pattern | Comes and goes repeatedly | VM | 28/33 | 46/71 | 85 | 35 | 38 | 83 |
| Temporal pattern | Continuous, all the time | PPPD | 13/33 | 6/71 | 39 | 92 | 68 | 76 |
| Duration | Days or longer | PPPD | 15/33 | 16/70 | 45 | 77 | 48 | 75 |
| Duration | Seconds to minutes | BPPV | 8/10 | 35/94 | 80 | 63 | 19 | 97 |

VM, vestibular migraine; PPPD, persistent postural-perceptual dizziness; BPPV, benign paroxysmal positional vertigo; PPV, positive predictive
