# Supplementary material for: What patients do when they feel dizzy: behavioural signatures reveal diagnostic clues in common vestibular disorders
Source: J Neurol. 2026 Sep 10;273(10):587. doi: 10.1007/s00415-026-14133-0 (PMC13562255; doi:10.1007/s00415-026-14133-0)
Supplement: Supplementary file 3 [file 415_2026_14133_MOESM3_ESM.docx]

**Supplementary Tables for provocative factors**

**Supplementary Table 4. Association Between Precipitating Factors and VM**

| **Variables in the Equation** | | | | | | | | | |
| --- | --- | --- | --- | --- | --- | --- | --- | --- | --- |
|  | | B | S.E. | Wald | df | Sig. | Exp(B) | 95% C.I.for EXP(B) | |
|  |  |  |  |  |  |  |  | Lower | Upper |
| Step 1^a^ | Q5a(1) | ,707 | ,568 | 1,549 | 1 | ,213 | 2,028 | ,666 | 6,175 |
|  | Q5b(1) | -,535 | ,534 | 1,002 | 1 | ,317 | ,586 | ,205 | 1,669 |
|  | Q5c(1) | -,644 | ,581 | 1,231 | 1 | ,267 | ,525 | ,168 | 1,638 |
|  | Q5d(1) | ,285 | ,594 | ,230 | 1 | ,632 | 1,329 | ,415 | 4,257 |
|  | Q5e(1) | 3,044 | ,614 | 24,598 | 1 | ,000 | 20,996 | 6,304 | 69,923 |
|  | Constant | -1,754 | ,572 | 9,396 | 1 | ,002 | ,173 |  |  |
| a. Variable(s) entered on step 1: Q5a, Q5b, Q5c, Q5d, Q5e. | | | | | | | | | |

Nagelkerke R² = 0.385, Hosmer–Lemeshow p = 0.253.

**Supplementary Table 5. Association Between Precipitating Factors and PPPD**

| **Variables in the Equation** | | | | | | | | | |
| --- | --- | --- | --- | --- | --- | --- | --- | --- | --- |
|  | | B | S.E. | Wald | df | Sig. | Exp(B) | 95% C.I.for EXP(B) | |
|  |  |  |  |  |  |  |  | Lower | Upper |
| Step 1^a^ | Q5a(1) | -1,248 | ,572 | 4,758 | 1 | ,029 | ,287 | ,094 | ,881 |
|  | Q5b(1) | ,304 | ,525 | ,334 | 1 | ,563 | 1,355 | ,484 | 3,793 |
|  | Q5c(1) | 2,522 | ,581 | 18,840 | 1 | ,000 | 12,451 | 3,987 | 38,881 |
|  | Q5d(1) | ,277 | ,578 | ,231 | 1 | ,631 | 1,320 | ,425 | 4,095 |
|  | Q5e(1) | -1,747 | ,694 | 6,344 | 1 | ,012 | ,174 | ,045 | ,679 |
|  | Constant | -1,263 | ,527 | 5,750 | 1 | ,016 | ,283 |  |  |
| a. Variable(s) entered on step 1: Q5a, Q5b, Q5c, Q5d, Q5e. | | | | | | | | | |

Nagelkerke R² = 0.350, Hosmer–Lemeshow p = 0.002.

**Supplementary Table 6. Association Between Precipitating Factors and BPPV**

| **Variables in the Equation** | | | | | | | | | |
| --- | --- | --- | --- | --- | --- | --- | --- | --- | --- |
|  | | B | S.E. | Wald | df | Sig. | Exp(B) | 95% C.I.for EXP(B) | |
|  |  |  |  |  |  |  |  | Lower | Upper |
| Step 1^a^ | Q5a(1) | 2,444 | 1,112 | 4,828 | 1 | ,028 | 11,518 | 1,302 | 101,886 |
|  | Q5b(1) | ,370 | ,678 | ,297 | 1 | ,585 | 1,448 | ,383 | 5,470 |
|  | Q5c(1) | -2,500 | 1,095 | 5,217 | 1 | ,022 | ,082 | ,010 | ,701 |
|  | Q5d(1) | -1,280 | 1,145 | 1,251 | 1 | ,263 | ,278 | ,029 | 2,620 |
|  | Q5e(1) | -19,362 | 6554,880 | ,000 | 1 | ,998 | ,000 | ,000 | . |
|  | Constant | -2,897 | 1,128 | 6,592 | 1 | ,010 | ,055 |  |  |
| a. Variable(s) entered on step 1: Q5a, Q5b, Q5c, Q5d, Q5e. | | | | | | | | | |

Nagelkerke R² = 0.434, Hosmer–Lemeshow p = 0.662.

**Supplementary Table 7. Association Between Precipitating Factors and OH**

| **Variables in the Equation** | | | | | | | | | |
| --- | --- | --- | --- | --- | --- | --- | --- | --- | --- |
|  | | B | S.E. | Wald | df | Sig. | Exp(B) | 95% C.I.for EXP(B) | |
|  |  |  |  |  |  |  |  | Lower | Upper |
| Step 1^a^ | Q5a(1) | -1,122 | ,696 | 2,596 | 1 | ,107 | ,326 | ,083 | 1,275 |
|  | Q5b(1) | 1,280 | ,687 | 3,470 | 1 | ,062 | 3,598 | ,935 | 13,836 |
|  | Q5c(1) | -1,414 | ,828 | 2,917 | 1 | ,088 | ,243 | ,048 | 1,232 |
|  | Q5d(1) | -,585 | ,750 | ,609 | 1 | ,435 | ,557 | ,128 | 2,423 |
|  | Q5e(1) | -19,407 | 7348,900 | ,000 | 1 | ,998 | ,000 | ,000 | . |
|  | Constant | -1,193 | ,658 | 3,289 | 1 | ,070 | ,303 |  |  |
| a. Variable(s) entered on step 1: Q5a, Q5b, Q5c, Q5d, Q5e. | | | | | | | | | |

Nagelkerke R² = 0.292, Hosmer–Lemeshow p = 0.714.

**Supplementary Table 8. Association Between Precipitating Factors and Structural Central Causes of Vertigo**

| **Variables in the Equation** | | | | | | | | | |
| --- | --- | --- | --- | --- | --- | --- | --- | --- | --- |
|  | | B | S.E. | Wald | df | Sig. | Exp(B) | 95% C.I.for EXP(B) | |
|  |  |  |  |  |  |  |  | Lower | Upper |
| Step 1^a^ | Q5a(1) | -,745 | ,798 | ,873 | 1 | ,350 | ,475 | ,099 | 2,266 |
|  | Q5b(1) | ,099 | ,777 | ,016 | 1 | ,899 | 1,104 | ,241 | 5,058 |
|  | Q5c(1) | -1,365 | 1,102 | 1,534 | 1 | ,216 | ,256 | ,029 | 2,215 |
|  | Q5d(1) | -1,544 | 1,166 | 1,753 | 1 | ,186 | ,214 | ,022 | 2,100 |
|  | Q5e(1) | -,589 | 1,122 | ,275 | 1 | ,600 | ,555 | ,062 | 5,007 |
|  | Constant | -1,380 | ,695 | 3,941 | 1 | ,047 | ,251 |  |  |
| a. Variable(s) entered on step 1: Q5a, Q5b, Q5c, Q5d, Q5e. | | | | | | | | | |

Nagelkerke R² = 0.127, Hosmer–Lemeshow p = 0.787.

**Supplementary Table 9. Association Between Precipitating Factors and Peripheral Vestibular Causes of Vertigo**

| **Variables in the Equation** | | | | | | | | | |
| --- | --- | --- | --- | --- | --- | --- | --- | --- | --- |
|  | | B | S.E. | Wald | df | Sig. | Exp(B) | 95% C.I.for EXP(B) | |
|  |  |  |  |  |  |  |  | Lower | Upper |
| Step 1^a^ | Q5a(1) | 2,208 | 1,490 | 2,197 | 1 | ,138 | 9,101 | ,491 | 168,791 |
|  | Q5b(1) | -20,623 | 4232,111 | ,000 | 1 | ,996 | ,000 | ,000 | . |
|  | Q5c(1) | 1,336 | 1,317 | 1,029 | 1 | ,310 | 3,804 | ,288 | 50,240 |
|  | Q5d(1) | 3,393 | 1,515 | 5,014 | 1 | ,025 | 29,747 | 1,526 | 579,711 |
|  | Q5e(1) | -18,749 | 6128,498 | ,000 | 1 | ,998 | ,000 | ,000 | . |
|  | Constant | -4,689 | 1,722 | 7,413 | 1 | ,006 | ,009 |  |  |
| a. Variable(s) entered on step 1: Q5a, Q5b, Q5c, Q5d, Q5e. | | | | | | | | | |

Nagelkerke R² = 0.525, Hosmer–Lemeshow p = 0.990.

**Supplementary Tables for Avoidance Behaviours**

**Supplementary Table 10. Avoidance Behaviours Associated with VM**

| **Variables in the Equation** | | | | | | | | | |
| --- | --- | --- | --- | --- | --- | --- | --- | --- | --- |
|  | | B | S.E. | Wald | df | Sig. | Exp(B) | 95% C.I.for EXP(B) | |
|  |  |  |  |  |  |  |  | Lower | Upper |
| Step 1^a^ | Q9a(1) | -1,240 | ,873 | 2,018 | 1 | ,155 | ,289 | ,052 | 1,601 |
|  | Q9b(1) | -,626 | ,596 | 1,105 | 1 | ,293 | ,535 | ,166 | 1,719 |
|  | Q9c(1) | ,819 | ,543 | 2,273 | 1 | ,132 | 2,269 | ,782 | 6,583 |
|  | Q9d(1) | ,805 | ,516 | 2,440 | 1 | ,118 | 2,237 | ,814 | 6,145 |
|  | Q9e(1) | -1,820 | ,637 | 8,176 | 1 | ,004 | ,162 | ,047 | ,564 |
|  | Q9f(1) | 2,105 | ,642 | 10,743 | 1 | ,001 | 8,203 | 2,330 | 28,875 |
|  | Constant | -1,098 | ,399 | 7,563 | 1 | ,006 | ,334 |  |  |
| a. Variable(s) entered on step 1: Q9a, Q9b, Q9c, Q9d, Q9e, Q9f. | | | | | | | | | |

Nagelkerke R² = 0.249, Hosmer–Lemeshow p = 0.586.

**Supplementary Table 11. Avoidance Behaviours Associated with PPPD**

| **Variables in the Equation** | | | | | | | | | |
| --- | --- | --- | --- | --- | --- | --- | --- | --- | --- |
|  | | B | S.E. | Wald | df | Sig. | Exp(B) | 95% C.I.for EXP(B) | |
|  |  |  |  |  |  |  |  | Lower | Upper |
| Step 1^a^ | Q9a(1) | 1,193 | ,641 | 3,463 | 1 | ,063 | 3,296 | ,938 | 11,579 |
|  | Q9b(1) | 1,515 | ,560 | 7,324 | 1 | ,007 | 4,550 | 1,519 | 13,632 |
|  | Q9c(1) | -,616 | ,579 | 1,132 | 1 | ,287 | ,540 | ,174 | 1,680 |
|  | Q9d(1) | -1,169 | ,531 | 4,848 | 1 | ,028 | ,311 | ,110 | ,880 |
|  | Q9e(1) | ,405 | ,566 | ,513 | 1 | ,474 | 1,500 | ,495 | 4,547 |
|  | Q9f(1) | -,013 | ,601 | ,000 | 1 | ,983 | ,987 | ,304 | 3,209 |
|  | Constant | -,992 | ,375 | 6,986 | 1 | ,008 | ,371 |  |  |
| a. Variable(s) entered on step 1: Q9a, Q9b, Q9c, Q9d, Q9e, Q9f. | | | | | | | | | |

Nagelkerke R² = 0.239, Hosmer–Lemeshow p = 0.562.

**Supplementary Table 12. Avoidance Behaviours Associated with BPPV**

| **Variables in the Equation** | | | | | | | | | |
| --- | --- | --- | --- | --- | --- | --- | --- | --- | --- |
|  | | B | S.E. | Wald | df | Sig. | Exp(B) | 95% C.I.for EXP(B) | |
|  |  |  |  |  |  |  |  | Lower | Upper |
| Step 1^a^ | Q9a(1) | -1,364 | 1,248 | 1,195 | 1 | ,274 | ,256 | ,022 | 2,950 |
|  | Q9b(1) | -1,248 | 1,154 | 1,169 | 1 | ,280 | ,287 | ,030 | 2,756 |
|  | Q9c(1) | -1,766 | ,914 | 3,736 | 1 | ,053 | ,171 | ,029 | 1,025 |
|  | Q9d(1) | 2,240 | ,857 | 6,828 | 1 | ,009 | 9,393 | 1,750 | 50,407 |
|  | Q9e(1) | ,910 | ,714 | 1,626 | 1 | ,202 | 2,485 | ,613 | 10,066 |
|  | Q9f(1) | -19,493 | 6678,787 | ,000 | 1 | ,998 | ,000 | ,000 | . |
|  | Constant | -2,779 | ,768 | 13,101 | 1 | ,000 | ,062 |  |  |
| a. Variable(s) entered on step 1: Q9a, Q9b, Q9c, Q9d, Q9e, Q9f. | | | | | | | | | |

Nagelkerke R² = 0.396, Hosmer–Lemeshow p = 0.980.

**Supplementary Table 13. Avoidance Behaviours Associated with OH**

| **Variables in the Equation** | | | | | | | | | |
| --- | --- | --- | --- | --- | --- | --- | --- | --- | --- |
|  | | B | S.E. | Wald | df | Sig. | Exp(B) | 95% C.I.for EXP(B) | |
|  |  |  |  |  |  |  |  | Lower | Upper |
| Step 1^a^ | Q9a(1) | -,531 | 1,110 | ,229 | 1 | ,632 | ,588 | ,067 | 5,178 |
|  | Q9b(1) | -1,195 | ,896 | 1,781 | 1 | ,182 | ,303 | ,052 | 1,751 |
|  | Q9c(1) | ,514 | ,669 | ,590 | 1 | ,442 | 1,672 | ,450 | 6,209 |
|  | Q9d(1) | -1,164 | ,672 | 3,002 | 1 | ,083 | ,312 | ,084 | 1,165 |
|  | Q9e(1) | 1,372 | ,698 | 3,863 | 1 | ,049 | 3,942 | 1,004 | 15,480 |
|  | Q9f(1) | -,848 | ,905 | ,878 | 1 | ,349 | ,428 | ,073 | 2,523 |
|  | Constant | -1,667 | ,491 | 11,517 | 1 | ,001 | ,189 |  |  |
| a. Variable(s) entered on step 1: Q9a, Q9b, Q9c, Q9d, Q9e, Q9f. | | | | | | | | | |

Nagelkerke R² = 0.163, Hosmer–Lemeshow p = 0.901.

**Supplementary Table 14. Avoidance Behaviours Associated with Structural Central Causes of Vertigo**

| **Variables in the Equation** | | | | | | | | | |
| --- | --- | --- | --- | --- | --- | --- | --- | --- | --- |
|  | | B | S.E. | Wald | df | Sig. | Exp(B) | 95% C.I.for EXP(B) | |
|  |  |  |  |  |  |  |  | Lower | Upper |
| Step 1^a^ | Q9a(1) | ,033 | 1,161 | ,001 | 1 | ,978 | 1,033 | ,106 | 10,056 |
|  | Q9b(1) | -18,228 | 6316,291 | ,000 | 1 | ,998 | ,000 | ,000 | . |
|  | Q9c(1) | -,094 | ,919 | ,010 | 1 | ,919 | ,910 | ,150 | 5,518 |
|  | Q9d(1) | -,286 | ,840 | ,116 | 1 | ,733 | ,751 | ,145 | 3,898 |
|  | Q9e(1) | ,777 | ,847 | ,840 | 1 | ,359 | 2,174 | ,413 | 11,444 |
|  | Q9f(1) | -18,055 | 6793,534 | ,000 | 1 | ,998 | ,000 | ,000 | . |
|  | Constant | -2,146 | ,611 | 12,335 | 1 | ,000 | ,117 |  |  |
| a. Variable(s) entered on step 1: Q9a, Q9b, Q9c, Q9d, Q9e, Q9f. | | | | | | | | | |

Nagelkerke R² = 0.198, Hosmer–Lemeshow p = 0.965.

**Supplementary Table 15. Avoidance Behaviours Associated with Peripheral Causes of Vertigo**

| **Variables in the Equation** | | | | | | | | | |
| --- | --- | --- | --- | --- | --- | --- | --- | --- | --- |
|  | | B | S.E. | Wald | df | Sig. | Exp(B) | 95% C.I.for EXP(B) | |
|  |  |  |  |  |  |  |  | Lower | Upper |
| Step 1^a^ | Q9a(1) | ,954 | 1,284 | ,553 | 1 | ,457 | 2,597 | ,210 | 32,170 |
|  | Q9b(1) | 1,136 | ,987 | 1,326 | 1 | ,250 | 3,115 | ,450 | 21,546 |
|  | Q9c(1) | ,934 | 1,039 | ,807 | 1 | ,369 | 2,544 | ,332 | 19,489 |
|  | Q9d(1) | ,833 | 1,058 | ,619 | 1 | ,431 | 2,299 | ,289 | 18,290 |
|  | Q9e(1) | -1,311 | 1,236 | 1,125 | 1 | ,289 | ,269 | ,024 | 3,041 |
|  | Q9f(1) | -18,923 | 7350,720 | ,000 | 1 | ,998 | ,000 | ,000 | . |
|  | Constant | -3,577 | 1,023 | 12,224 | 1 | ,000 | ,028 |  |  |
| a. Variable(s) entered on step 1: Q9a, Q9b, Q9c, Q9d, Q9e, Q9f. | | | | | | | | | |

Nagelkerke R² = 0.190, Hosmer–Lemeshow p = 0.943.

**Supplementary Tables for Behavioural Responses**

**Supplementary Table 16. Behavioural Responses Associated with VM**

| **Variables in the Equation** | | | | | | | | | |
| --- | --- | --- | --- | --- | --- | --- | --- | --- | --- |
|  | | B | S.E. | Wald | df | Sig. | Exp(B) | 95% C.I.for EXP(B) | |
|  |  |  |  |  |  |  |  | Lower | Upper |
| Step 1^a^ | Q10a(1) | 1,774 | ,524 | 11,466 | 1 | ,001 | 5,897 | 2,111 | 16,469 |
|  | Q10b(1) | -,259 | ,512 | ,257 | 1 | ,613 | ,772 | ,283 | 2,104 |
|  | Q10c(1) | -,306 | ,474 | ,416 | 1 | ,519 | ,737 | ,291 | 1,866 |
|  | Q10d(1) | ,383 | ,480 | ,635 | 1 | ,425 | 1,467 | ,572 | 3,761 |
|  | Q10e(1) | ,766 | ,476 | 2,584 | 1 | ,108 | 2,150 | ,845 | 5,469 |
|  | Q10f(1) | ,010 | ,547 | ,000 | 1 | ,986 | 1,010 | ,345 | 2,953 |
|  | Constant | -1,515 | ,526 | 8,293 | 1 | ,004 | ,220 |  |  |
| a. Variable(s) entered on step 1: Q10a, Q10b, Q10c, Q10d, Q10e, Q10f. | | | | | | | | | |

Nagelkerke R² = 0.210, Hosmer–Lemeshow p = 0.073.

**Supplementary Table 17. Behavioural Responses Associated with PPPD**

| **Variables in the Equation** | | | | | | | | | |
| --- | --- | --- | --- | --- | --- | --- | --- | --- | --- |
|  | | B | S.E. | Wald | df | Sig. | Exp(B) | 95% C.I.for EXP(B) | |
|  |  |  |  |  |  |  |  | Lower | Upper |
| Step 1^a^ | Q10a(1) | -,314 | ,580 | ,293 | 1 | ,588 | ,730 | ,234 | 2,278 |
|  | Q10b(1) | -,267 | ,490 | ,297 | 1 | ,586 | ,766 | ,293 | 1,999 |
|  | Q10c(1) | ,373 | ,472 | ,624 | 1 | ,430 | 1,452 | ,575 | 3,663 |
|  | Q10d(1) | -,901 | ,467 | 3,716 | 1 | ,054 | ,406 | ,162 | 1,015 |
|  | Q10e(1) | -,172 | ,481 | ,127 | 1 | ,721 | ,842 | ,328 | 2,164 |
|  | Q10f(1) | 1,238 | ,499 | 6,164 | 1 | ,013 | 3,448 | 1,298 | 9,162 |
|  | Constant | -,781 | ,510 | 2,343 | 1 | ,126 | ,458 |  |  |
| a. Variable(s) entered on step 1: Q10a, Q10b, Q10c, Q10d, Q10e, Q10f. | | | | | | | | | |

Nagelkerke R² = 0.184, Hosmer–Lemeshow p = 0.898.

**Supplementary Table 18. Behavioural Responses Associated with BPPV**

| **Variables in the Equation** | | | | | | | | | |
| --- | --- | --- | --- | --- | --- | --- | --- | --- | --- |
|  | | B | S.E. | Wald | df | Sig. | Exp(B) | 95% C.I.for EXP(B) | |
|  |  |  |  |  |  |  |  | Lower | Upper |
| Step 1^a^ | Q10a(1) | -19,799 | 7764,183 | ,000 | 1 | ,998 | ,000 | ,000 | . |
|  | Q10b(1) | 1,866 | ,741 | 6,339 | 1 | ,012 | 6,461 | 1,512 | 27,607 |
|  | Q10c(1) | -,876 | ,673 | 1,693 | 1 | ,193 | ,416 | ,111 | 1,558 |
|  | Q10d(1) | ,123 | ,679 | ,033 | 1 | ,857 | 1,131 | ,299 | 4,283 |
|  | Q10e(1) | -,027 | ,725 | ,001 | 1 | ,970 | ,974 | ,235 | 4,030 |
|  | Q10f(1) | -1,118 | 1,132 | ,974 | 1 | ,324 | ,327 | ,036 | 3,009 |
|  | Constant | -2,123 | ,860 | 6,088 | 1 | ,014 | ,120 |  |  |
| a. Variable(s) entered on step 1: Q10a, Q10b, Q10c, Q10d, Q10e, Q10f. | | | | | | | | | |

Nagelkerke R² = 0.314, Hosmer–Lemeshow p = 0.473.

**Supplementary Table 19. Behavioural Responses Associated with OH**

| **Variables in the Equation** | | | | | | | | | |
| --- | --- | --- | --- | --- | --- | --- | --- | --- | --- |
|  | | B | S.E. | Wald | df | Sig. | Exp(B) | 95% C.I.for EXP(B) | |
|  |  |  |  |  |  |  |  | Lower | Upper |
| Step 1^a^ | Q10a(1) | -1,423 | 1,122 | 1,608 | 1 | ,205 | ,241 | ,027 | 2,174 |
|  | Q10b(1) | -,765 | ,656 | 1,357 | 1 | ,244 | ,466 | ,129 | 1,685 |
|  | Q10c(1) | ,388 | ,626 | ,384 | 1 | ,536 | 1,474 | ,432 | 5,028 |
|  | Q10d(1) | ,963 | ,663 | 2,110 | 1 | ,146 | 2,619 | ,714 | 9,604 |
|  | Q10e(1) | ,654 | ,637 | 1,057 | 1 | ,304 | 1,924 | ,553 | 6,699 |
|  | Q10f(1) | -1,793 | 1,110 | 2,610 | 1 | ,106 | ,166 | ,019 | 1,465 |
|  | Constant | -2,091 | ,775 | 7,276 | 1 | ,007 | ,124 |  |  |
| a. Variable(s) entered on step 1: Q10a, Q10b, Q10c, Q10d, Q10e, Q10f. | | | | | | | | | |

Nagelkerke R² = 0.196, Hosmer–Lemeshow p = 0.820.

**Supplementary Table 20. Behavioural Responses Associated with Structural Sentral Causes of Vertigo**

| **Variables in the Equation** | | | | | | | | | |
| --- | --- | --- | --- | --- | --- | --- | --- | --- | --- |
|  | | B | S.E. | Wald | df | Sig. | Exp(B) | 95% C.I.for EXP(B) | |
|  |  |  |  |  |  |  |  | Lower | Upper |
| Step 1^a^ | Q10a(1) | -18,721 | 7253,563 | ,000 | 1 | ,998 | ,000 | ,000 | . |
|  | Q10b(1) | -1,052 | ,885 | 1,412 | 1 | ,235 | ,349 | ,062 | 1,980 |
|  | Q10c(1) | -,541 | ,786 | ,473 | 1 | ,492 | ,582 | ,125 | 2,720 |
|  | Q10d(1) | -,107 | ,819 | ,017 | 1 | ,896 | ,898 | ,181 | 4,469 |
|  | Q10e(1) | -1,136 | 1,127 | 1,017 | 1 | ,313 | ,321 | ,035 | 2,920 |
|  | Q10f(1) | -19,037 | 6814,825 | ,000 | 1 | ,998 | ,000 | ,000 | . |
|  | Constant | -,912 | ,879 | 1,075 | 1 | ,300 | ,402 |  |  |
| a. Variable(s) entered on step 1: Q10a, Q10b, Q10c, Q10d, Q10e, Q10f. | | | | | | | | | |

Nagelkerke R² = 0.275, Hosmer–Lemeshow p = 0.841.

**Supplementary Table 21. Behavioural Responses Associated with Peripheral Vestibular Causes of Vertigo**

| **Variables in the Equation** | | | | | | | | | |
| --- | --- | --- | --- | --- | --- | --- | --- | --- | --- |
|  | | B | S.E. | Wald | df | Sig. | Exp(B) | 95% C.I.for EXP(B) | |
|  |  |  |  |  |  |  |  | Lower | Upper |
| Step 1^a^ | Q10a(1) | 1,237 | 1,052 | 1,383 | 1 | ,240 | 3,445 | ,439 | 27,065 |
|  | Q10b(1) | 1,380 | ,972 | 2,015 | 1 | ,156 | 3,975 | ,591 | 26,709 |
|  | Q10c(1) | 1,818 | 1,227 | 2,193 | 1 | ,139 | 6,157 | ,555 | 68,268 |
|  | Q10d(1) | -,739 | ,972 | ,578 | 1 | ,447 | ,478 | ,071 | 3,209 |
|  | Q10e(1) | -19,526 | 5881,382 | ,000 | 1 | ,997 | ,000 | ,000 | . |
|  | Q10f(1) | ,248 | 1,015 | ,060 | 1 | ,807 | 1,281 | ,175 | 9,361 |
|  | Constant | -4,323 | 1,291 | 11,211 | 1 | ,001 | ,013 |  |  |
| a. Variable(s) entered on step 1: Q10a, Q10b, Q10c, Q10d, Q10e, Q10f. | | | | | | | | | |

Nagelkerke R² = 0.297, Hosmer–Lemeshow p = 0.964.

**Supplementary Table 22. Univariable associations between temporal characteristics (Question 2) and diagnostic groups.**

| **Question** | **Answer Category** | **Diagnosis** | **OR (95% CI)** | **P value** |
| --- | --- | --- | --- | --- |
| Q2 | Suddenly, has happened once | VM | 1.75 (0.55-5.53) | 0.340 |
| Q2 | Comes and goes repeatedly | VM | 3.04 (1.04-8.86) | 0.041 |
| Q2 | Continuous, all the time | VM | 0.20 (0.04-0.94) | 0.042 |
| Q2 | Suddenly, has happened once | PPPD | 0.31 (0.04-1.50) | 0.149 |
| Q2 | Comes and goes repeatedly | PPPD | 0.40 (0.17-0.97) | 0.043 |
| Q2 | Continuous, all the time | PPPD | 7.04 (2.36-20.93) | < 0.001 |
| Q2 | Suddenly, has happened once | BPPV | 0.69 (0.08-5.92) | 0.737 |
| Q2 | Comes and goes repeatedly | BPPV | 4.64 (0.56-38.29) | 0.144 |
| Q2 | Continuous, all the time | BPPV | N/A | N/A |
| Q2 | Suddenly, has happened once | OD | 0.98 (0.19-4.93) | 0.983 |
| Q2 | Comes and goes repeatedly | OD | 2.03 (0.53-7.76) | 0.299 |
| Q2 | Continuous, all the time | OD | 0.28 (0.03-2.28) | 0.236 |
| Q2 | Suddenly, has happened once | CSVD | 0.91 (0.10-8.03) | 0.932 |
| Q2 | Comes and goes repeatedly | CSVD | 1.23 (0.23-6.29) | 0.803 |
| Q2 | Continuous, all the time | CSVD | 0.61 (0.07-5.35) | 0.663 |
| Q2 | Suddenly, has happened once | PVD | 4.83 (0.73-31.93) | 0.102 |
| Q2 | Comes and goes repeatedly | PVD | 0.08 (0.01-0.83) | 0.034 |
| Q2 | Continuous, all the time | PVD | 3.21 (0.49-20.73) | 0.219 |

VM, vestibular migraine; PPPD, persistent postural-perceptual dizziness; BPPV, benign paroxysmal positional vertigo; OD, orthostatic dizziness; CSVD, central structural vestibular disorder(s); PVD, peripheral vestibular disorder(s); OR, odds ratio; CI, confidence interval.

**Supplementary Table 22. Univariable associations between dizziness duration (Question 3) and diagnostic groups.**

| **Question** | **Answer Category** | **Diagnosis** | **OR (95% CI)** | **P value** |
| --- | --- | --- | --- | --- |
| Q3 | Seconds to minutes | VM | 0.64 (0.27-1.52) | 0.319 |
| Q3 | Minutes to hours | VM | 1.45 (0.60-3.50) | 0.401 |
| Q3 | Days or longer | VM | 1.25 (0.51-3.04) | 0.623 |
| Q3 | Seconds to minutes | PPPD | 0.53 (0.22-1.27) | 0.156 |
| Q3 | Minutes to hours | PPPD | 0.78 (0.31-1.95) | 0.599 |
| Q3 | Days or longer | PPPD | 2.81 (1.16-6.80) | 0.022 |
| Q3 | Seconds to minutes | BPPV | 6.74 (1.35-33.56) | 0.020 |
| Q3 | Minutes to hours | BPPV | 0.533 (0.10-2.66) | 0.444 |
| Q3 | Days or longer | BPPV | N/A | N/A |
| Q3 | Seconds to minutes | OD | 1.76 (0.58-5.29) | 0.312 |
| Q3 | Minutes to hours | OD | 1.61 (0.52-4.99) | 0.405 |
| Q3 | Days or longer | OD | 0.13 (0.01-1.10) | 0.062 |
| Q3 | Seconds to minutes | CSVD | 1,46 (0.34-6.19) | 0.607 |
| Q3 | Minutes to hours | CSVD | 1.38 (0.31-6.18) | 0.669 |
| Q3 | Days or longer | CSVD | 0.31 (0.03-2.62) | 0.283 |
| Q3 | Seconds to minutes | PVD | 0.94 (0.15-5.89) | 0.950 |
| Q3 | Minutes to hours | PVD | N/A | N/A |
| Q3 | Days or longer | PVD | 3.75 (0.59-23.66) | 0.160 |

VM, vestibular migraine; PPPD, persistent postural-perceptual dizziness; BPPV, benign paroxysmal positional vertigo; OD, orthostatic dizziness; CSVD, central structural vestibular disorder(s); PVD, peripheral vestibular disorder(s); OR, odds ratio; CI, confidence interval.
